# Extracts from the Records of the Boston Society for Medical Improvement

**Published:** 1851-11

**Authors:** William W. Moreland

**Affiliations:** Secretary


ARTICLE IV.

*Extracts from the Records of the Boston Society for Medical Improvement.* By WM. W. MORLAND, M. D., Secretary.

*May* 12. *Hydrocephalus.*—Dr. COALE reported the case of A. H., born January 14th, 1849. Parents healthy ; first child. Dr. C. was called to see her when she was two weeks old ; found her laboring under cerebral symptoms, which soon resolved themselves into undeniable signs of water on the brain. She was treated with small doses of calomel, and afterwards with hydriodate of potassa, with apparent benefit at first. The head, however, steadily increased in size. The general health was good except when disturbed by teething—at which time she had occasional spasms, never amounting, however, to a general convulsion.

| | Measurement of head. | Inches. | | Inches. |
|---|---|---|---|---|
| Sept. 12th, 1849. | Over crown from meatus to meatus | 12½ | Round | 18½ |
| Nov. 1st, " | " " " " " " | 13½ | " | 19½ |
| " 28th, " | " " " " " " | 14 | " | 20 |
| May 10th, 1351. | " " " " " " | 17¾ | " | 23½ |

The family having moved out of town in Aug. 1850, Dr. C. did not see the child after that except at rare periods. The last time was May 10th, 1851. Her height is now thirty-one inches; she lies on her back ; is blind, but hears, though imperfectly. The pressure above has forced down the vault of the orbit so that the eyeball seems lower, and more covered by the lower than the upper lid ; much of the white above the cornea being exposed, whilst the cornea is half covered by the lower lid. The mouth contains the usual number of teeth. Motion of limbs perfect, but feeble, except of right arm, which is paralyzed almost entirely. Fond of throwing the left hand about, and with it occasionally feels the right arm, and resists any attempts to meddle with it. Extremities cold, making it necessary to keep a good fire in the room day and night through the winter. Never cries or frets. Takes, three times a day, ten ounces of milk, sucked from a bottle. Bowels open with regularity once a day.

The child died two months and a half after this, without any remarkable change.

*May* 26. *Otorrhœa of Twenty Years' Duration, terminating fatally from Hemorrage.* Case furnished by Dr. F. H. Gray. Dr. Parkmean showed the specimen.—F. C., twenty-one years of age, of scrofulous habit, though having a good share of health, had been troubled with a purulent discharge of fetid character from the right ear, from infancy.

On the evening of April 10th, 1851, patient rode several miles on horseback, and on the following morning complained of general uneasiness, though sufficiently able to attend to his ordinary business. On the morning of the 15th, severe pain commenced in right ear, which continued for three successive days, at the end of which period, copious and offensive purulent discharges found their way into the meatus auditorius and likewise into the mouth. Patient was greatly relieved by the discharges, and was able to walk and ride out, though he still suffered from headache, until the morning of the 21st, when some coagulated blood was ejected from the mouth. Copious hemorrhage took place from the ear into the mouth at intervals, varying in quantity from ℥iv to Oj, during the next twenty hours, when he quietly laid himself back, and expired. During the whole illness, there was an almost daily occurrence of vomiting, with the pulse unusually slow, possibly to be referred to the influence of narcotics.

At the autopsy, there was found a slight bloody effusion in the lower surface of the cerebellum, proceeding from a small gangrenous opening in the posterior surface of the right lateral sinus, just before it terminates in the jugular vein ; the sinus was also ulcerated on the side next the petrous portion of the temporal bone, and blood was extensively effused into the cavity of the ear and into the cellular tissue behind the pharynx. The petrous portion of the temporal bone, sawn through and exhibited, showed the cavity of the ear deeply affected with caries, and undoubtedly the inflammation had spread from this point, involving the sinus. The specimen is in the Society's Cabinet.—*Amer. Jour. Med. Sci.*

